# Biodiversity and Activity of the Gut Microbiota across the Life History of the Insect Herbivore *Spodoptera littoralis*

**DOI:** 10.1038/srep29505

**Published:** 2016-07-08

**Authors:** Bosheng Chen, Beng-Soon Teh, Chao Sun, Sirui Hu, Xingmeng Lu, Wilhelm Boland, Yongqi Shao

**Affiliations:** 1 College of Animal Sciences, Zhejiang University, 310058 Hangzhou, China; 2 Department of Bioorganic Chemistry, Max Planck Institute for Chemical Ecology, Beutenberg Campus, D-07745 Jena, Germany; 3 Institute of Biotechnology, Zhejiang University, 310058 Hangzhou, China.

## Abstract

Microbes that live inside insects play critical roles in host nutrition, physiology, and behavior. Although Lepidoptera (butterflies and moths) are one of the most diverse insect taxa, their microbial symbionts are little-studied, particularly during metamorphosis. Here, using ribosomal tag pyrosequencing of DNA and RNA, we investigated biodiversity and activity of gut microbiotas across the holometabolous life cycle of *Spodoptera littoralis*, a notorious agricultural pest worldwide. Proteobacteria and Firmicutes dominate but undergo a structural “metamorphosis” in tandem with its host. *Enterococcus*, *Pantoea* and *Citrobacter* were abundant and active in early-instar, while *Clostridia* increased in late-instar. Interestingly, only enterococci persisted through metamorphosis. Female adults harbored high proportions of *Enterococcus*, *Klebsiella* and *Pantoea*, whereas males largely shifted to *Klebsiella*. Comparative functional analysis with PICRUSt indicated that early-instar larval microbiome was more enriched for genes involved in cell motility and carbohydrate metabolism, whereas in late-instar amino acid, cofactor and vitamin metabolism increased. Genes involved in energy and nucleotide metabolism were abundant in pupae. Female adult microbiome was enriched for genes relevant to energy metabolism, while an increase in the replication and repair pathway was observed in male. Understanding the metabolic activity of these herbivore-associated microbial symbionts may assist the development of novel pest-management strategies.

Insects are colonized by various microorganisms, and with the development of next-generation sequencing (NGS) technologies, a rapidly growing body of work, particularly on bees, ants and flies, has shown that these symbiotic associates have important effects on host nutrition, development and pathogen defense^1^,^2^,^3^,^4^,^5^. For example, the prevalence of bacterial gut symbionts, *Rhizobiales*, is tightly linked with the evolution of herbivory of ants, which supply additional nitrogen to the host^6^. Abundant lactic acid bacteria, maintained in biofilms in the foregut of Western honeybees (*Apis mellifera*), work in a synergistic manner to inhibit the proliferation of pathogens^7^. The phytophagous Lepidopterans, including butterflies and moths, are one of the most widespread and diverse taxa of insects on our planet, containing about 160,000 described species in 47 superfamilies^8^, and are also major pests in agriculture; however, surprisingly, their associated microbial symbionts have not been studied much with modern molecular tools, making it difficult to identify the potential impacts that these microbes may have on host ecology and evolution.

Lepidopteran insects are holometabolous and develop through four life stages; each stage has its own morphology. The egg hatches into a larva, which feeds, molts and grows larger, pupates, then emerges as an adult insect that looks completely different from the larva. Currently most studies of the lepidopteran microbiota focus on microorganisms associated with the larval gut, providing only a single snapshot of the community. For example, conventional culture-dependent techniques have identified several proteolytic bacteria, including *Enterococcus spp.* and *Bacillus spp.*, from the gut of the velvetbean caterpillar *Anticarsia gemmatalis*^9^. Culture-independent techniques have revealed a core microbial community in the larval gut of the cotton leafworm, *Spodoptera littoralis*^10^. In contrast, almost nothing is known about the diversity and composition of microbial communities inhabiting other stages, in particular, the adult microbiota; nor it is known how microbial populations may change over metamorphosis^11^. To address these gaps in our knowledge, in the present study we used a high-throughput sequencing-based approach to compare the structure of the bacterial community in replicate egg masses, larvae, pupae and adults of *S. littoralis*, a highly polyphagous lepidopteran pest found worldwide and also an important model system used in a variety of biological research^12^. To our knowledge, this is the first systematic survey of bacterial communities across the full life cycle of a moth species, providing a foundation for future studies of microbial symbiosis in this important insect group.

In addition to investigating how the *S. littoralis*-associated microbial community varies with life stages, we also aimed to determine the metabolically active populations within the community. Despite a proliferation of studies that document the 16S rRNA gene profiles of gut communities, few address whether taxa that are detected in the DNA pool are actually active cells (DNA extracted from samples can include DNA from dead or dominant bacteria, and extracellular DNA from lysed or degraded cells, which in fact do not have any metabolic activity.). While 16S rRNA from the RNA pool represents protein synthesis potential and can be used as an indicator of active microbes, which directly contribute to the current function of the microbiota. Thus the 16S rRNA/rRNA gene phylotype ratio (RNA/DNA) is, in principle, a measure of relative activity. Many studies have employed this methodology to characterize the active moiety of a microbial community from diverse environments^13^,^14^,^15^,^16^. For instance, Reid *et al*. used this method to evaluate the diversity of metabolically active and inactive bacteria in the wood-feeding beetle larval gut^15^. A comparison of the 16S rRNA gene versus 16S rRNA-derived data sets revealed that *Prochlorococcus* spp. play a more important role in the food web of oligotrophic sea than expected^16^. Taking advantage of the NGS technology, here we fully assessed the *S. littoralis* microbiota by examining both the DNA-based 16S rRNA gene and the RNA (cDNA)-based 16S rRNA with pyrosequencing, an ideal tool for exploring the vast majority and often uncultivable microbes in complex microbial communities. The recently developed software package, Phylogenetic Investigation of Communities by Reconstruction of Unobserved States (PICRUSt), was further used to delineate metabolic potentials of the organism represented by 16S rRNA sequence, based on the phylogenetic placement of that 16S rRNA sequence within a phylogeny of sequenced genomes, an approach demonstrated previously^17^. These comprehensive analyses of 16S rRNA sequences were expected to provide insights that have not been revealed by past studies into the total diversity and metabolic activity of Lepidoptera-associated microbial communities.

## Results

### Overview of *Spodoptera littoralis* microbiotas

**Figure 1:**
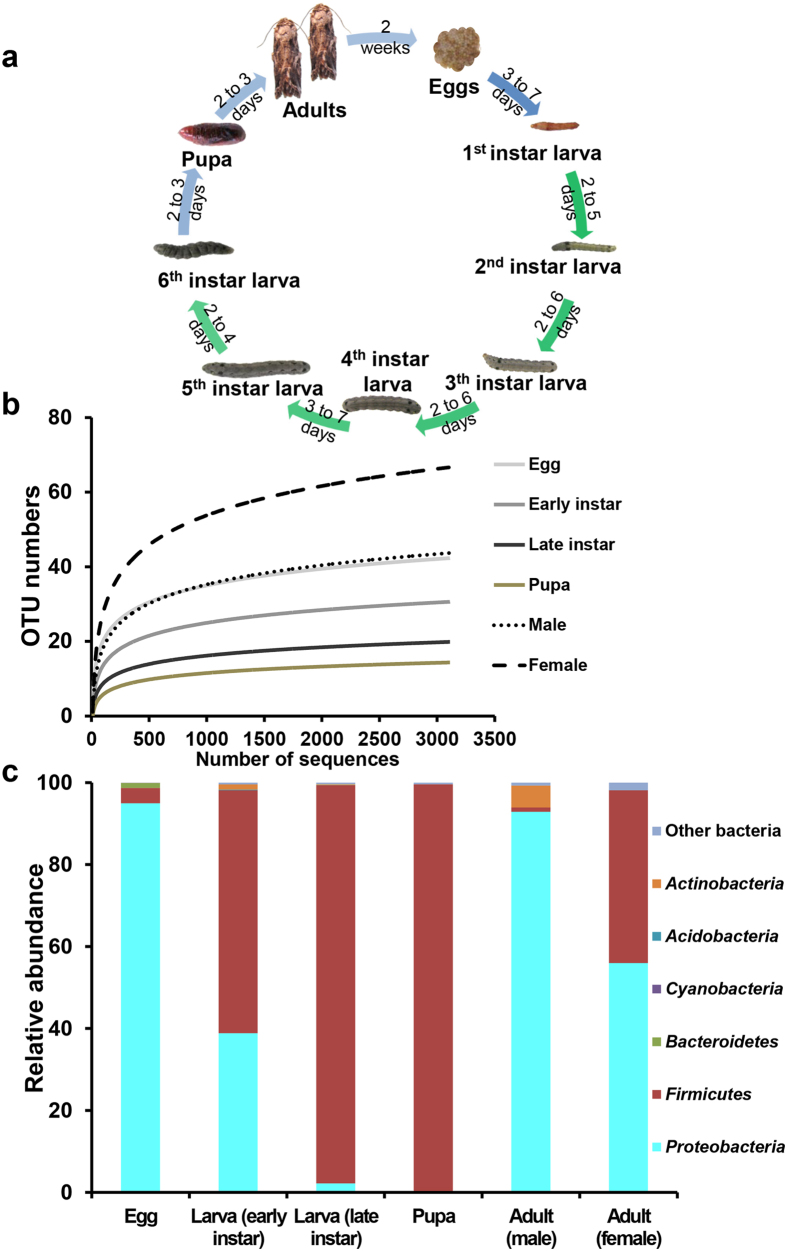
Changes in bacterial community diversity across life stages of *S. littoralis*. (**a**) Overview of development stages of the host. (**b**) Rarefaction curves depicted from randomly subsampled data sets with the same number of 16S sequences. The near saturated rarefaction curve indicates that the vastness of microbial diversity was retrieved from each sample. (**c**) Overview of the microbiota change during host development. Abundance of the 16S rRNA gene at each developmental stage at the phylum level. Relative abundances of the most dominant phyla including Proteobacteria, Firmicutes and Actinobacteria changed significantly (*p* < 0.001) across the life cycle.

Like all Lepidoptera, *S. littoralis* undergoes complete metamorphosis; larvae and adults are greatly differentiated in form and function (Fig. 1a). Larvae chew on plant leaves while adults often feed on nectar. Our study divided the relatively long larval stage into early-instar and late-instar stages; adults live for only a few days after eclosion. Neither culturing on nutrient agar plates nor PCR amplification using specific primers revealed any fungus or Archaea in any of our samples. While we found abundant bacteria persisting in all developmental stages of *S. littoralis*, the colony-forming unit (CFU) counts in the larva, pupa and adult were 6.3 × 10^7^, 1.02 × 10^4^, and 2.81 × 10^5^ per sample, respectively. However, the diversity and composition of the *S. littoralis*-associated bacterial community (designated as microbiota) varies substantially across host developmental stages.

The microbial community in the egg masses was more diverse (43 OTUs per sample, analyzed at a 3% dissimilarity level) than the community identified in the early-instar larvae (34 OTUs per sample). Bacterial phylotype richness further decreased to 23 in late-instar larvae (Table 1). Pupae were associated with the lowest number of phylotypes, demonstrating an increasing reduction in the microbial diversity from egg to pupa. This trend was also true for Shannon diversity, phylotype evenness and phylogenetic diversity, all of which displayed similar patterns (Table 1 and Supplementary figure S1). In contrast, adults harbored high richness of bacteria with 73 and 46 phylotypes in females and males, respectively. The gradually flattening rarefaction curves confirmed that the vast majority of microbial diversity was captured in all samples (Fig. 1b).

A taxonomic analysis of sequences obtained by pyrosequencing revealed that the most prevalent phylum in the microbial community associated with *S. littoralis* egg masses was Proteobacteria (ca. 95% of the sequences), whereas the most prevalent phylum in the larvae was Firmicutes (ca. 59% of the sequences in the early-instar vs. 97% in the late-instar) (Fig. 1c). Notably, through metamorphosis, pupae also harbored a bacterial community rich in Firmicutes (ca. 99% of sequences). After eclosion, we further observed a remarkable change in the composition of the bacterial community. Mature adults, especially males, exhibited a large decrease in the relative abundances of Firmicutes and showed an increased abundance of Proteobacteria (Fig. 1c). The adult male gut microbiota was dominated by Proteobacteria (ca. 93% of the sequences), together with Actinobacteria (5%), whereas the adult female gut microbiota consisted of 56% Proteobacteria and 42% Firmicutes, respectively. Relative abundances of the most highly represented phyla including Proteobacteria, Firmicutes and Actinobacteria, changed significantly (*p* < 0.001) across life stages. The pattern of taxon distribution in each stage is described in detail in the following sections.

**Figure 2:**
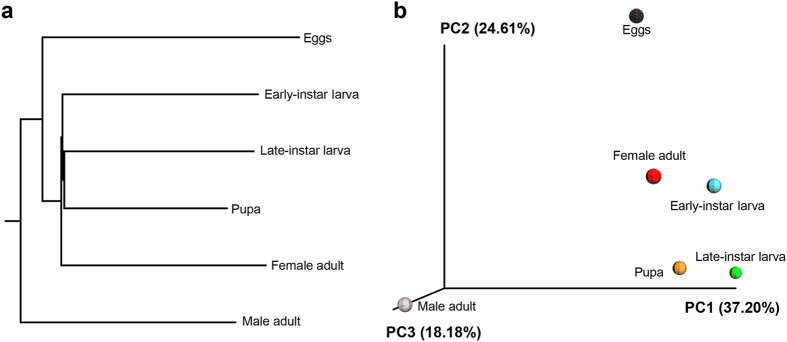
Similarity analysis of microbial communities. (**a**) UPGMA clustering of samples at different developmental stages according to community composition and structure. (**b**) Principal coordinates analysis (PCoA) plot visualizing the data based on β-diversity metrics of UniFrac.

We compared community structures between samples using principal coordinate analysis (PCoA). Pairwise ecologic distances were calculated based on the β-diversity metrics of weighted and unweighted UniFrac, which takes into account both community membership and relatedness of community members^18^. After sequence jackknifing, these distances then were visualized by the Emperor PCoA plot, which displayed the similarity among communities (Fig. 2).

### Variability of community composition within individuals

**Figure 3:**
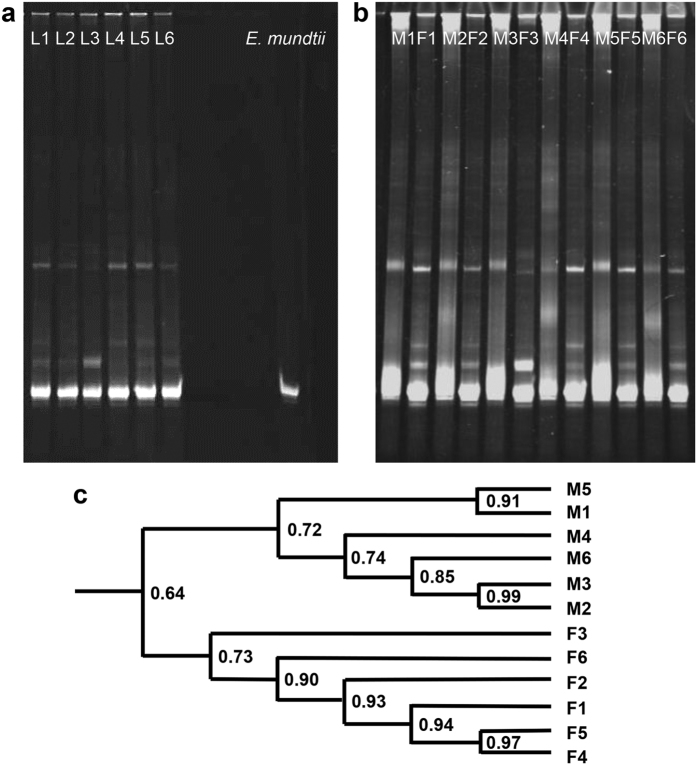
DGGE profiles of PCR-amplified 16S rRNA gene fragments of bacterial communities from *S. littoralis* larva and adult samples. (**a**) DGGE profile of the mature larval gut microbiota of different individuals (L = larva). (**b**) DGGE profile of the adult gut microbiota of different individuals (M = male adult and F = female adult). (**c**) Cluster analysis of the DGGE patterns of the male (M) and female (F) samples.

**Figure 4:**
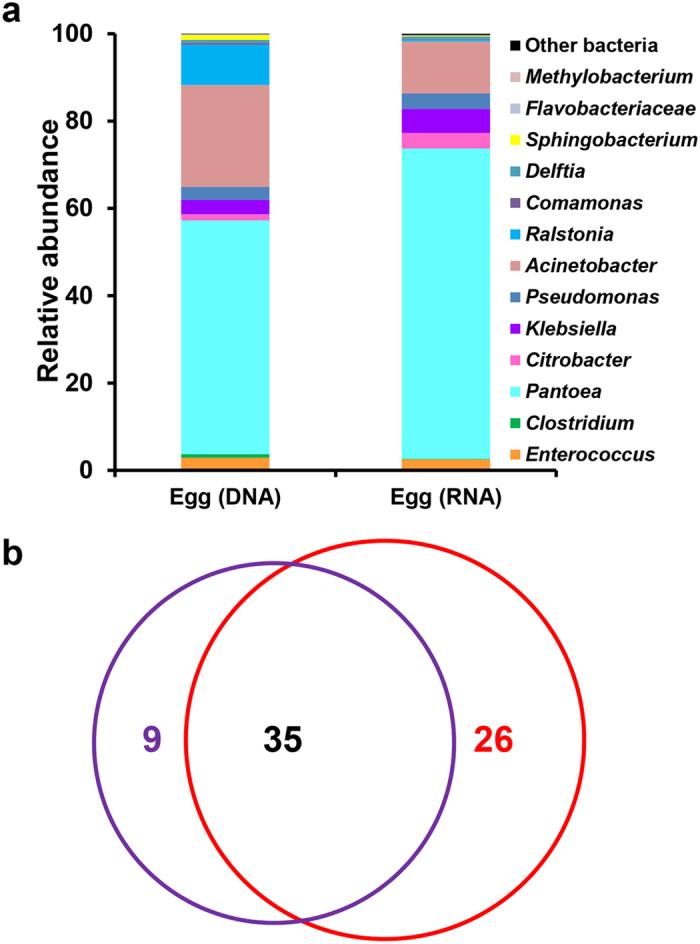
The microbiota associated with the egg mass of *S. littoralis*. (**a**) Relative abundance of major taxa (to genera level) in the DNA and RNA data sets. (**b**) Venn diagram showing overlaps of OTUs (at 97% similarity) between the DNA (purple circle) and RNA (red circle) data sets. Values are the numbers of OTUs calculated using the total data set.

**Figure 5:**
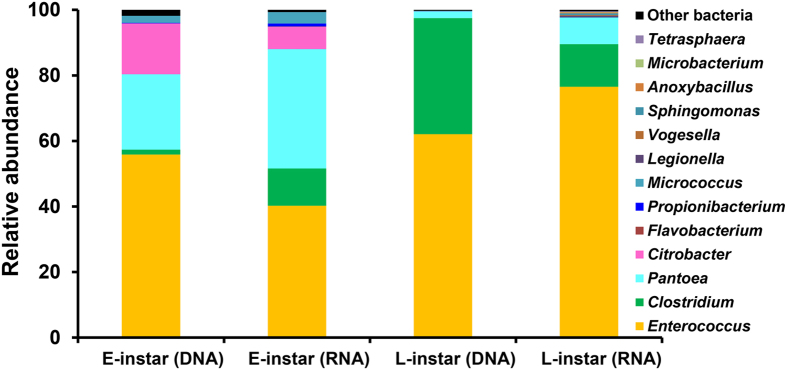
The larval gut microbiota of *S. littoralis*. Relative abundance of major taxa (to genera level) in the DNA and RNA data sets of early-instar larvae (E-instar) and late-instar larvae (L-instar).

**Figure 6:**
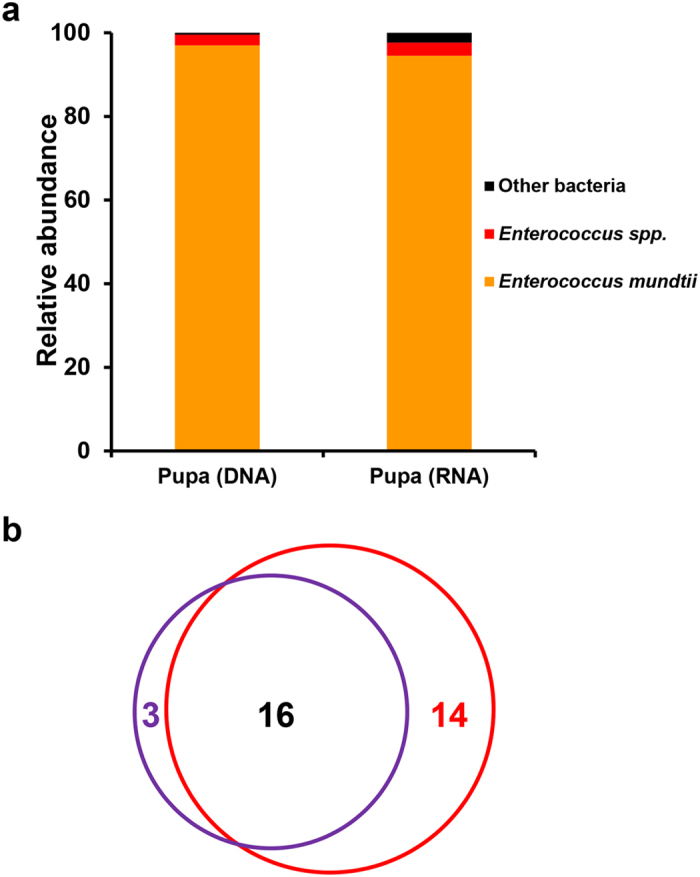
The microbiota associated with the pupae of *S. littoralis*. (**a**) Relative abundance of major taxa in the DNA and RNA data sets. (**b**) Venn diagram showing overlaps of OTUs (at 97% similarity) between the DNA (purple circle) and RNA (red circle) data sets.

**Figure 7:**
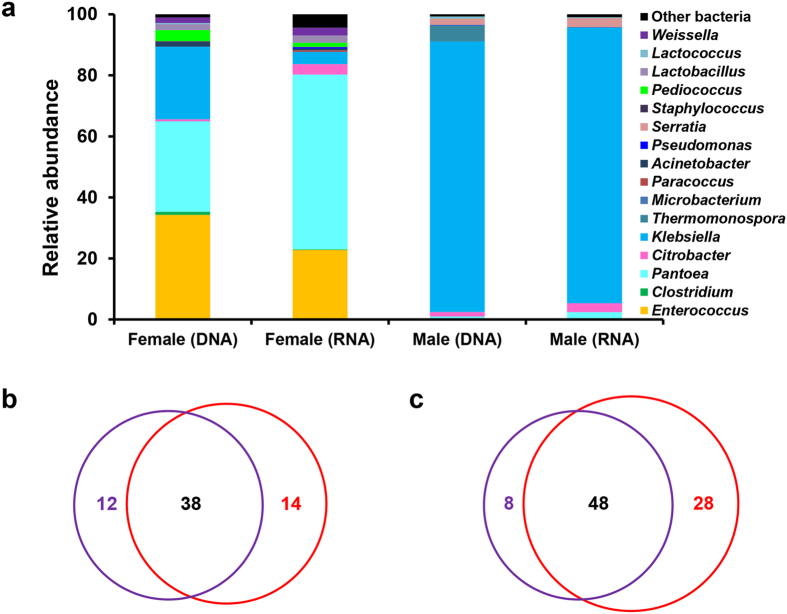
The adult gut microbiota of *S. littoralis*. (**a**) Relative abundance of major taxa (to genera level) in the DNA and RNA data sets of female and male adults. (**b**) Venn diagram showing overlaps of OTUs (at 97% similarity) between the DNA (purple circle) and RNA (red circle) data sets of male adult and female adult (**c**).

**Figure 8:**
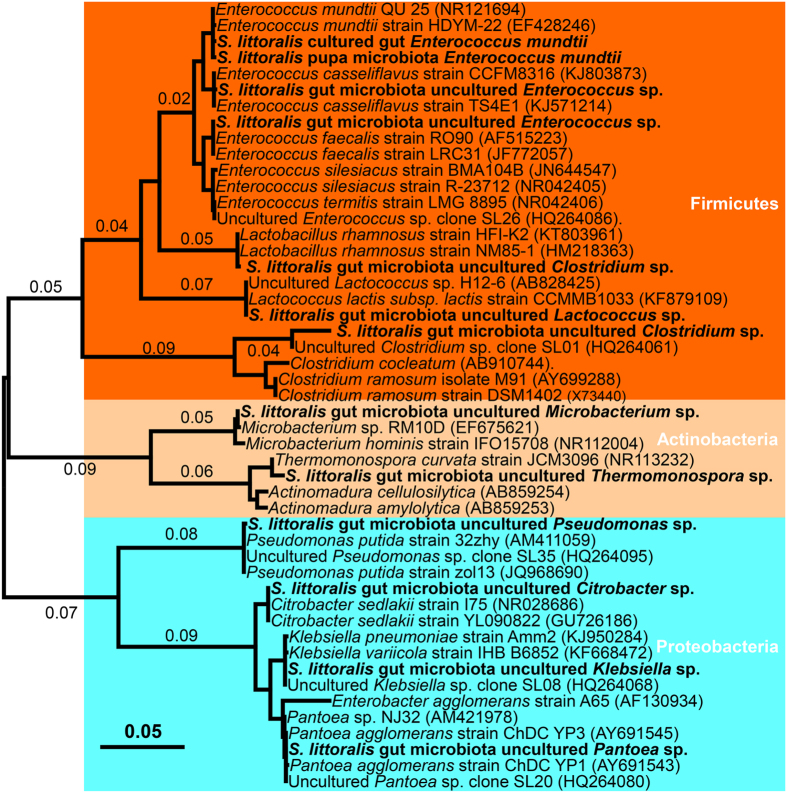
Phylogenetic analysis of dominant taxa identified from microbiotas associated with *S. littoralis*. Maximum-likelihood tree constructed on the basis of 16S rRNA gene sequences. Bootstrap values were obtained from a search with 500 replicates. Strain and accession numbers are given behind the species names.

We first evaluated the variability of community composition among individuals by using denaturing gradient gel electrophoresis (DGGE) of amplified 16S rRNA genes, a commonly used molecular technique for rapid fingerprint analysis of the microbial community. A cultured gut bacterium, *Enterococcus mundtii*, representing the most prevalent taxon isolated from *S. littoralis* in our previous study, was used as a DGGE standard. The DGGE profile showed that there was little variation among different individuals of a laboratory population reared under identical environmental conditions (Fig. 3a, larvae; b, adults). Similar DGGE band profiles indicated similar patterns of microbial community structure and diversity. The DGGE pattern of the larval gut microbiota was rather simple, consisting mostly of *E. mundtii* (the intense band, Fig. 3a). Likewise, similar DGGE band profiles were also observed among adults, both females and males. Although specific bacterial taxa existed in both populations, a more diverse microbial community was observed among females and a substantial difference was observed between female and male fingerprints (Fig. 3b). The three dominant bands in the female samples were weaker or absent in the male samples. The clustering analysis of DGGE profiles clearly differentiated female from male samples (Fig. 3c). The results from PCR-DGGE analysis showed that larvae subjected to the same conditions at the same life stage harbored communities that were highly similar in structure and membership, whereas among adults, the communities differed between males and females. Massive parallel pyrosequencing of the 16S was subsequently used to provide detailed taxonomic information (Figs 4, 5, 6, 7, 8).

### Egg microbiota

*S. littoralis* lays a batch of eggs, not single eggs, on the leaves of plants, where the eggs are exposed to a wide range of environmental microbes. Eggs are attached to each other tightly and are covered with hair-like scales derived from the tip of the abdomen of the female moth (Fig. 1a and Supplementary video S1). A complex community of bacteria, dominated by the Proteobacteria, was associated with the egg masses. Large number of 16S sequences that were obtained by pyrosequencing was classified to the genus taxonomic level. The bacterial taxa were largely members of the genus *Pantoea* (53.5%), *Acinetobacter* (23.4%) and *Ralstonia* (9.2%), all belonging to the Proteobacteria (Fig. 4a). Other phylotypes within this phylum, classified as *Citrobacter*, *Klebsiella* and *Pseudomonas*, were also observed in the egg mass. Only a minor fraction of sequences were identified as *Enterococcus* (2.9%) and *Clostridium* (0.8%) belonging to the Firmicutes. Bacteria from other phyla, such as the genus *Sphingobacterium* in the Bacteroidetes phylum, were discovered in the egg samples too, but many taxa were present in low proportions (less than 0.1%).

The results from the RNA (cDNA)-based 16S rRNA data reflected the metabolically active bacterial populations, i.e., those with higher ribosomal content, suggesting candidates which may play key roles *in situ*. *Pantoea* occupied the highest relative abundance (71.1%) in the active microbial community (Fig. 4a). *Citrobacter*, *Klebsiella* and *Pseudomonas* were also more abundant in the RNA data set, implying their high activity. In contrast, *Ralstonia* dominated in the DNA data set (9.2%) but not in the RNA data set (0.4%), indicating low metabolic activity on the egg surface. *Acinetobacter* was also less abundant in the RNA data. Unsurprisingly, the strictly anaerobic *Clostridia* did not show activity since the egg was exposed to the air. These bacteria were present but not functioning *in situ*. The variation in bacterial composition between the DNA and RNA data sets was characterized using a Venn diagram (Fig. 4b). Overall, 35 OTUs were shared between groups. These shared OTUs represented the majority of sequences, indicating that the major phylotypes were metabolically active. There were more unique OTUs in the RNA data set (26) than in the DNA data set (9). However, most of these phylotypes were rare members of the community, representing <0.01% sequences in the total data set.

We observed that before the neonate larvae hatch out from the egg, they have already started feeding inside and have to bite enough eggshell material to make a hole before they can escape from the egg case (Supplementary video S1). Thus the microbiota associated with egg mass represents the maternal and environmental sources of gut bacteria.

### Larval microbiota

**Figure 9:**
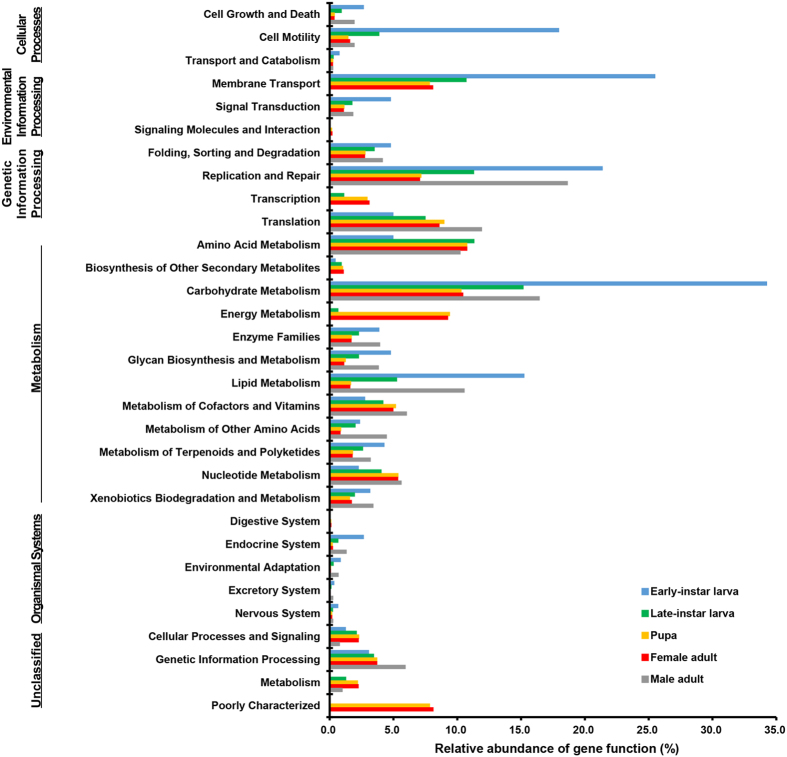
Inferred functions of bacterial communities associated with *S. littoralis*. All of the predicted KEGG metabolic pathways are shown at the second hierarchical level and grouped by major functional categories.

Upon hatching, the neonate larva starts feeding and develops. Most bacteria associated with the eggmass were also observed in the early-instar larval gut microbiota, indicating gut symbionts are acquired by newborn hosts from the mother via egg. However, relative abundances of bacterial taxa differed largely between the microbiota of starting egg mass and that of larval gut. The taxonomic composition of the gut microbiota of early 2^nd^ instar revealed a relatively low abundance of Proteobacteria sequences (approximately 38.9% of all sequences), in contrast to the higher level on the egg mass (95%). *Pantoea* decreased to just 23% in the larval gut (Fig. 5). Another dominant Proteobacteria that was closely related to *Citrobacter* comprised 15.6% of the community. In contrast, although only a minor fraction of *Enterococcus* was associated with the egg mass, it was particularly high in abundance (55.9%) in the larval gut. These major phylotypes were detected in both DNA and RNA data sets (Fig. 5). Although a low abundance of *Clostridium* was found in the DNA data set, it was one of dominant members of the RNA-derived fraction, indicating its high metabolic activity inside the gut. The Firmicutes appeared to be replacing Proteobacteria as larvae developed. Taking advantage of PICRUSt, we predicted functional potentials of the microbial community associated with different developmental stages. The early-instar larval gut microbiome was more enriched for genes involved in cell motility, carbohydrate metabolism and transport pathways (Fig. 9). From a functional standpoint, the enrichment of these pathways could have a number of implications.

The gut microbiota consistently changed with host development. Late-instar larvae harbored a community with largely lower species diversity than that in early-instars. The Firmicutes flourished within the larval gut. *Enterococcus* was the most stable component in the microbiota, representing 62.1% of all sequences (Fig. 5). Similarly, a higher proportion (35.4%) of the gut bacterial community was found to belong to the *Clostridium* genus. On the contrary, members of the Proteobacteria phylum were significantly cleared from the larval gut microbiota; *Pantoea* was the exception, present in both stages. Each dominant genus also had a large fraction in the RNA-based data set. Functional differences were also observed in bacterial populations associated with different developmental stages. A relative increase in genes associated with amino acid, cofactor and vitamin metabolism pathways was observed in late-instar larvae (Fig. 9). Overall, despite the complex microbial diversity associated with the egg mass, the larval gut community became highly simplified through host development. The resulting gut communities were similar to each other within the population (Fig. 3a).

### Metamorphosis

Each fully grown larva forms a cylindrical pupa (Fig. 1a). During metamorphosis, the overall body organization of the larva changes completely: most organs undergo deep remodeling or even completely degenerate, and differentiation processes are required to form the new body structure typical of the adult insect. The pupae show the presence of bacteria, and such bacterial populations seem to have no negative effect on traits related to the fitness of *S. littoralis*. However, the bacterial diversity in the pupal stage, indicated by the Shannon index, dropped to 0.26 from 1.46 in the late-larval stage, and 0.06 by the Simpson index (Table 1). Only enterococci dominated both RNA and DNA data sets, and they made up more than 97% of the sequences in both data sets (Fig. 6). Representative sequences of every *Enterococcus* OTU were separated from other sequences and compared with those available in the GenBank databases for more accurate identification. The representative of the most abundant OTU was identified as *E. mundtii* (Fig. 8). Several functional categories, such as those associated with carbohydrate metabolism, lipid metabolism, signal transduction, and membrane transport, diminished to 50% or more in the internal bacterial community of the pupa. Whereas genes involved in energy and nucleotide metabolism, transcription and translation pathways were abundant in the pupa, indicating a greater ability of the bacterial population to extract energy for surviving inside. After sampling, all the pupae successfully emerged and developed into adults.

### Adult microbiota

After eclosion, adult moths were maintained under identical conditions for mating and fed on sugar solution because of their usual nectivorous lifestyle. The mature adults successfully mated and produced a normal amount of eggs. In light of the difference between male and female adults in their reproductive physiology and degree of nectar feeding, the internal bacterial communities associated with mature adults of each sex were studied separately. Overall, significant recolonization of the gut had been observed in adults and the richness of microbial species was restored (Table 1). However, a clear structural change in bacterial community was identified between larva and adult, although enterococci prevailed into adulthood through metamorphosis. Phylotypes belonging to the bacterial family *Enterobacteriaceae* were overrepresented in adults relative to larvae. Furthermore, female and male adult gut microbiotas differed greatly in terms of the relative proportion of the most abundant bacteria each harbored (Fig. 7).

The bacterial community in female adults had a greater diversity relative to that in male adults. Besides *Enterococcus* occupied a considerable proportion (34.3%), other Firmicutes, including *Weissella*, *Pediococcus*, *Clostridium* and *Lactobacillus* spp., were also found in female adults (Fig. 7a). *Pantoea* were also well established in the gut flora, representing 29.7% of all sequences, while only a small proportion of *Citrobacter* was identified. The Gram-negative bacterium *Klebsiella* sp. was another prominent enterobacteria in the DNA data set (23.8%) but not in the RNA data set (4.0%). Main active bacterial taxa included *Pantoea*, *Enterococcus*, and *Citrobacter*. Interestingly, these bacteria were also found in the microbiota associated with the eggmass, indicating that certain bacteria might be transmitted to the filial generation. Venn diagrams showed that there were more OTUs in the RNA data set (Fig. 7b and c), but these phylotypes represented a low proportion of the total amount (less than 0.1%), indicating that rare phylotypes contributed to the metabolic processes in the gut.

Compared to female adults, male adults harbored a much higher proportion of microbes belonging to the familiar *Enterobacteriaceae*. Although *Pantoea* largely decreased in the male gut flora, *Klebsiella* sp. was particularly abundant in the male sample, representing >88% of all sequences, with the remaining percentage being made up of *Thermomonospora*, *Serratia* and *Citrobacter*. A similar profile was observed in the RNA data set, revealing that most taxa were active inside the gut. However, the Firmicutes, like *Enterococcus*, were maintained at a very low level in the males compared to in the females.

Using PICRUSt, we identified significant differences between the functional potentials of the bacterial community compositions (Fig. 9). These functional categories, including energy metabolism, membrane transport, transcription and biosynthesis of secondary metabolites, were enriched in the female adults, whereas in the male adults, the replication and repair pathway and associated relative gene copy numbers were increased by approximately 50%. The enrichment of several other pathways, including those for cell growth and death, and lipid metabolism, was observed in the male microbiome but not in the female.

Considering the robustness of enterococci across all stages of the host’s development, we further screened the field-collected *S. littoralis* insects with the *Enterococcus*-specific primer. Field populations of *S. littoralis* were frequently associated with *E. mundtii* (Table 2), reflecting the significant role played by *E. mundtii* in host biology.

## Discussion

Although accumulating studies have described the microbial diversity in the insect gut, to date there have been few reports comparing metabolic activities in the microbial populations associated with successive life stages. In the present work, we not only conducted microbial inventories of *S. littoralis* across the full host life cycle by sequencing the 16S rRNA gene, but we also systematically investigated metabolically active bacteria by evaluating 16S rRNA contents, which provide new insights into metabolic potentials of moth-associated microbial communities.

In general, a large proportion of OTUs (87%, representing 95% of sequences) were active within samples, indicating the host gut is a “hot spot” for diverse microbial activities. This result concurs with a previous study on the gut flora of wood-feeding huhu beetles (*Prionoplus reticularis*, Cerambycidae) that showed many bacterial phylotypes are active^15^. However, not all active bacteria could successfully colonize inside the host. Despite high diverse in starting egg mass, a significant reduction in bacterial diversity was observed during the development of *S. littoralis* from egg to pupa, highlighting the control the host has over its gut microbiota (Fig. 1). In addition, individuals subjected to the standard rearing conditions at the same developmental stages harbored communities that were highly conserved in structure and membership (Fig. 3). Overall, the microbiota of *S. littoralis* exhibits low phylum-level diversity compared to the microbiota of the wood-feeding termite or of the beetle *Odontotaenius disjunctus*^19^,^20^. Only a few bacterial species, mainly belonging to the phyla Firmicutes and Proteobacteria, were detected from *S. littoralis*, yet it is consistent with previous reports describing the low species richness of the microbiota in other lepidopterans. For example, tobacco hornworm, *Manduca sexta*, harbored a rather simple gut microflora consisting mostly of phylotypes belonging to *Enterococcus*^21^. A similar midgut bacterial community was revealed in the larvae of the gypsy moth (*Lymantria dispar* L.) fed on different diets, and distinct from its foliar diet^22^. The physiological and biochemical conditions within the host insect’s alimentary tract appear to play an important role in structuring these communities. It is recognized that a straight alimentary canal contains fewer microorganisms. The extremely high pH (>10) in the lepidopteran larval gut could also act as a distinct selection pressure on microbial composition^23^. Considering that Lepidoptera are highly phytophagous insects, and the larval stage is most devastating, ingesting large amounts of plant materials and other potentially harmful microbes associated with their food, it makes sense for the host to efficiently control its gut microbiota and quickly clear invading microbes from its habitat. Therefore, the lepidopteran larval gut is a strongly selective eco-environment for its microbiome, and it may be common for larvae to maintain a relatively simple gut microbiota.

Furthermore, a developmental change in the most abundant species, from *Pantoea* and *Citrobacter* (Proteobacteria) in young larvae to *Enterococcus* and *Clostridium* (Firmicutes) in matured larvae was identified. All these dominant taxa are frequently detected in lepidopteran species. In particular, the *Enterococcus* genus successfully occupied the ecological niche and stably colonized the larval gut, despite its numerical inferiority in the egg microbiota. *Enterococci* have been found to be the most common gut bacteria in Lepidoptera, both wild and laboratory-reared populations^24^,^25^,^26^. For instance, *Enterococci* have been identified in the tobacco hornworm (Lepidoptera: Sphingidae), the gypsy moth (Lepidoptera: Erebidae), as well as the velvetbean caterpillar (Lepidoptera: Noctuidae), suggesting these bacteria perform some conserved functions in this highly phytophagous insect. Large amounts of *Citrobacter*, a genus within the *Enterobacteriaceae* family, occurred in the neonate larvae. Although this bacterium is known to form host associations with a variety of insects^21^, its biological relevance remains unclear. *Pantoea*, another highly versatile and diverse enterobacteria, have been isolated from many environments^27^ and consist of taxa with known capacities for degrading and utilizing different types of plant materials. As such, *Pantoea*, are putatively helpful bacteria for herbivores. *Clostridium* emerged as dominant commensals only in the mature larval gut. In the late instar, the larval gut exhibits a prevailing anoxic atmosphere, which favors the development of anaerobic microorganisms, such as *Clostridia*, and facultative anaerobic enterococci^10^. This might be the dominant force influencing the shift of gut microflora composition from Proteobacteria to Firmicutes.

This difference in taxonomic membership may reflect divergent functional roles across particular life stages. Analyses of metabolic activity, based on the RNA, suggested these taxa actively function *in vivo* (Fig. 5). PICRUSt builds a predictive understanding of the functions of these symbionts within the host (Fig. 9). The gut microbiome was significantly enriched for genes involved in the carbohydrate metabolism pathway. The dominant *Gammaproteobacteria* in the family *Enterobacteriaceae* are well equipped to degrade major structural components of plant materials. *Pantoea* spp. can produce diverse enzymes, including β-galactosidases (GH2), α-xylosidases (GH31), α-mannosidases (GH47), and α-rhamnosidases (GH78), as well as pectinesterases (CE8) involved in the plant polymer degradation^28^. It has been discovered that *Citrobacter amalonaticus* is capable of breaking down chitin, reflecting the metabolic diversity of *Gammaproteobacteria*. These Proteobacteria symbionts could play similar functions in *S. littoralis* and might be important nutrient providers for host insects in their early life stages. Much research in insects and other animals has shown that increases in the Firmicutes are related to an increased ability to harvest energy from the diet. *Clostridia* species such as *C. thermocellum* and *C. ljungdahlii* are known to have a robust capacity to degrade cellulose and hemicellulose, and to metabolize amino acids^29^. The presence of a large proportion of *Clostridia* is likely to be important for efficient biomass utilization. Therefore, those bacterial symbionts likely also play important roles in nutrition. Data from PICRUSt is further supported by previous work, which employed comparative genomic analysis of the microbiome of the cutworm *Agrotis ipsilon* (Lepidoptera: Noctuidae)^30^. The predominance of *Enterococcus* and its high metabolic activity suggest that this bacterium has a functional significance with regard to its host. As members of the gypsy moth gut flora, enterococci have been shown to prevent colonization by pathogens^31^. In this study, we found that genes involved in the metabolism of terpenoids and polyketides are consistently expressed in the *S. littoralis* microbiome (Fig. 9). The isolated *E. mundtii* symbionts also have the ability to produce antimicrobials. Thus the dominant *E. mundtii* bacteria are most likely to be defensive mutualists. Altogether, the characteristic gut microbiota found in *S. littoralis* larvae may provide various benefits to the lepidopteran host ranging from nutrient supplementation to host defense.

Lepidopterans are holometabolous, and the transition from larvae to adult is a metabolically dynamic and complex process. The host gut microbiota also undergoes significant structural changes during metamorphosis and in the adult stage. Although the gut during the transition from larvae to adult is believed to undergo sterilization process and adults recruit new microbiota^32^, it is interesting to observe here that *Enterococcus* species, mostly *E. mundtii*, are able to survive the metamorphosis and be transmitted to the emerged adults (Figs 6 and 7). Genes involved in energy and nucleotide metabolism, transcription and translation pathways were enriched in this enterococcal population. However, their exact roles inside the pupa are not understood and warrant further investigation.

The adult lepidopteran microbiota remains largely unexplored. There have been no previous culture-independent studies of microbial communities associated with adult moths. Because of their nectivorous lifestyle, the adult moth typically has a small and morphologically distinct gut in contrast to that of the larva. We found that *S. littoralis* adults host relatively complex bacterial communities, and the microbial community structure of the female adult differs from that of the male (Fig. 7). Firmicutes, mostly enterococci, formed a significant proportion of the female adult gut microbiota, while those bacteria remained at low levels in male adults. *Pantoea* and *Klebsiella* were another dominant taxa in female adults, whereas only *Klebsiella* was observed in male adults. Both *Pantoea* and *Klebsiella* belong to the Proteobacteria family *Enterobacteriaceae*, which occur widely in the guts of Lepidoptera and other herbivores and are potentially beneficial, nonpathogenic microbes^28^,^33^. Using level 2 KEGG predictions of ortholog function, differences between the functional potentials of the bacterial communities were also observed. The female adult microbiome was enriched for genes relevant to energy metabolism, while in the male adult, an increase in the replication and repair pathway was detected.

We know from studies on other holometabolous insect groups that adults may have similar microbiotas^34^, or different microbiotas as larvae^35^, or have sexually dimorphic microbiotas^36^. For example, the gut of adult cockchafer beetle *Melolontha hippocastani* housed the same microbial species that were present in the larval midgut, despite having metamorphosed from larva to beetle^34^. In contrast, a developmental change in the most abundant gut bacteria was identified in the fruitfly *Drosophila melanogaster*^35^. Notably, the bacterial composition of adult black flies *Simulium* spp. differed between males and females although they were collected from the same habitat^36^. Similarly, *Klebsiella* sp. was demonstrated to be relatively high in adult males of *Anopheles stephensi* but was not found in larvae and pupae^37^. Sexually dimorphic phenomena of the associated bacterial communities have also been reported in other animals. For example, female Antarctic seals harbor more Firmicutes in the gut, while males have more Fusobacteria^38^. Although the reasons for this shift are not well understood, several factors, including the radical change of internal physicochemical conditions in the digestive tract, host immune responses and disturbance might underlie this difference.

Interestingly, major taxa associated with the female adult, such as *Enterococcus*, were also detected in the eggmass, and these taxa further colonized the larval gut, suggesting that some gut symbionts are probably vertically transmitted. The maternal transmission of the core gut microbiota to the next generation might stabilize host-microbe interactions and facilitate co-evolution.

Recently, several comparative genomic and metagenomic studies of lepidopteran species have revealed an ancient and intimate relationship between bacteria and lepidopteran herbivores. It is reported that a gene encoding the enzyme that detoxifies plant-produced cyanide did not evolve in Lepidoptera but was horizontally transferred from bacteria^39^. Clearly, the gut microbiota is an important source for diverse microbial activities and a “hot spot” for microbe-host interactions. A better understanding of the relationship of microbial symbionts to the lepidopteran host would lead to new concepts and approaches to control insect pests by manipulating their microbiota. Additionally, *S. littoralis* provides an attractive model for exploring complex microbial symbioses, as it has a simplified gut structure and microbial community, and is now genetically amenable^40^. The current study helps advance our understanding of ecological and evolutionary roles of gut symbionts in an important insect group.

## Methods

### Insect rearing, field collections and sample processing

*S. littoralis* larvae were hatched from eggs and reared on artificial diet as previously described^41^. Plastic cabinets with the diet and the larvae were kept at 23–25 °C under a regime of 16 h illumination and a dark period of 8 h. The emerged adults were supplied with a sucrose solution. The field population was collected from a vegetable gardening area in the vicinity of Hangzhou, China, in August 2015. The egg masses, larvae, pupae and adults were transported to the laboratory in Petri dishes and kept at −20 °C prior to dissection. The eggs’ hatching process was recorded by a video camera.

For sample processing, all insects were first rinsed three times in sterile water, surface-sterilized in 70% ethanol for 30 s and rinsed again in sterile water. The whole gut tissue was dissected from each individual and homogenized for nucleic acid extraction, as previously described^41^. After dissection, the typical vitellogenic ovariole was observed in the mature female. The whole surface-sterilized pupa was used to investigate the internal bacteria. Egg masses were not surface-sterilized. The processed samples were first aseptically homogenized in 500 μL of sterile PBS. A serial dilution of 10-fold was performed by transferring 100 μL of the homogenized sample into 900 μL PBS, vortexing vigorously, and spread-plating 100 μL of each dilution onto Brain-heart infusion agar plates (8130, BD). All plates were incubated at 37 °C for 48 h. Total bacterial cells were counted as colony forming units (CFUs) for each sample.

### Nucleic acid extraction and reverse transcription

The dissected insect tissues (*n* = 6 at each stage) were first ground under liquid N_2_ with single-use, Eppendorf tube-adapted sterile pestles and then directly incubated with nucleic acid extraction solution, according to the manufacturer’s protocols (MC85200, Epicentre) with minor modifications. An additional lysozyme incubation step (30 min at 37 °C) was included to break up Gram-positive bacterial cells. The quality of extracted total nucleic acid was checked on the agarose gel and quantified using a NanoDrop 1000 (Thermo Scientific). DNA and RNA were further purified from the extracted total nucleic acid following manufacturer’s guidelines.

Extracted RNA was reverse-transcribed into cDNA using the QuantiTect Reverse Transcription kit (205311, Qiagen) according to the manufacturer’s guidelines. RNA was first treated with genomic DNA Wipeout buffer at 42 °C for 2 min to eliminate any trace of co-extracted DNA. A volume of 7 μL of the DNase-treated RNA was used for reverse transcription to cDNA in a total reaction volume of 10 μL using random primers. Two negative controls were performed, including 7 μL of DNase-treated RNA with all RT reagents except for the reverse transcriptase and 7 μL of RT-PCR grade water instead of RNA.

### Denaturing gradient gel electrophoresis (DGGE) of amplified 16S rRNA genes

PCR primers 968F/1401R were used to amplify the V6-V8 portion of 16S rRNA genes as previously described^26^. Archaea- and fungus-specific primers were used to amplify archaeal 16S and fungus ITS genes, respectively (Supplementary table S1)^42^,^43^. DGGE analysis was performed using the Bio-Rad DCode system. Electrophoresis was done using a 16 × 16 cm, 1 mm thick gel that contained 8% polyacrylamide with a 20 to 80% denaturant gradient (100% denaturant was 7 M urea and 40% (v/v) deionized formamide). The gels were run at 100 V for 16 h at 60 °C in TAE buffer (40 mM Tris–acetate, 1 mM EDTA; pH 7.4). After electrophoresis, the gels were stained for 30 min in TAE buffer with SYBR-Gold nucleic acid gel stain (S-11494, Invitrogen) for photographing. Gels were scanned using a GS-800 calibrated densitometer (Bio-Rad). Analysis of DGGE profiles (band match and clustering) was carried out using Quantity One software (version 4. 6.1; Bio-Rad), as described previously^44^.

### Pyrosequencing, data analysis and PCR screen

Bacterial tag-encoded FLX amplicon pyrosequencing (bTEFAP) was performed using a Roche 454 FLX instrument with Titanium reagents as described previously^41^. Basically, the hypervariable V1–V3 segment in the 16S rDNA was amplified using the fusion primer set Gray28F (5-GAGTTTGATCNTGGCTCAG-3) and Gray519r (5-GTNTTACNGCGGCKGCTG-3) extended with the respective primer Adaptor A/B and sample-specific multiplex identifiers (MID). The sequencing library was generated through one-step PCR with 30 cycles, using a Hot Start High Fidelity Taq Polymerase (Qiagen). Amplicons were sequenced based on the supplier protocol (Research and Testing Laboratory, Lubbock, TX, USA, http://www.researchandtesting.com). The reads extended from the forward direction (Gray28F), and all low-quality reads (quality cut-off = 25) and sequences <200 bp in length were removed following sequencing (Supplementary table S2).

The software package Quantitative Insight into Microbial Ecology (QIIME, 1.6.0 version) was used to process sequencing data and to calculate diversity^45^. Sequences first underwent quality control to remove potential artifacts and errors (the denoise_wrapper.py script in QIIME was used in our analysis) and trimmed of the part with low quality^46^. Chimera (detection method: ChimeraSlayer) and low abundance reads (<0.1%) were further removed from analysis^47^. Cdhit and uclust with 97% similarity cut-offs were used in multiple OTU picking to cluster the high-quality reads into operational taxonomic units (OTUs). For each OTU, the most abundant sequence was extracted as a representative sequence for each OTU picked and aligned to the Greengenes core set (http://greengenes.lbl.gov/) using PyNAST with the minimum sequence identity percent set to 75%^48^. The RDP classifier was employed to determine the highest resolution of taxonomy based on the Ribosomal Database Project (http://rdp.cme.msu.edu/). Finally, an OTU table was generated describing the occurrence of bacterial phylotypes within the sample. Representative sequences were aligned to reference sequences obtained from the NCBI nucleotide database using the ClustalW algorithm. UniFrac was used for microbial community comparison according to Lozupone *et. al*^18^. Shared and unique OTUs are graphically represented in Venn diagrams described elsewhere^15^. The identification of OTUs that were significantly different in abundance was carried out in METASTATS using the nonparametric t-test against the taxonomic data extracted from QIIME^49^. The significance level to threshold (P value) was set at 0.05. Phylogenetic trees were calculated using the Maximum Likelihood method (Tamura-Nei model) with 500 bootstrap replicates in MEGA5^50^.

To generate a synthetic metagenome, the observed 16S rDNA sequences were clustered into a collection of OTUs using the pick closed reference otus.py script in QIIME. The resultant biom-formatted OTU table was first normalized with respect to inferred 16S rRNA gene copy numbers and then used to predict metagenomic functional content based on the software package Phylogenetic Investigation of Communities by Reconstruction of Unobserved States (PICRUSt)^17^. This computational approach exploits the relationship between phylogeny and function by combining 16s data with a database of reference genomes (Greengenes) to predict the presence of gene families. Functional predictions were exported as KEGG orthologs.

To show that *E. mundtii* is associated with the field population of *S. littoralis*, a primer specific for *E. mundtii* was utilized to screen for the symbiont using diagnostic PCR reactions (Table 2)^51^. PCR amplifications were conducted on a Mastercycler Gradient Thermocycler (Eppendorf, Germany) using 20 μL reactions, including 1 μL of DNA template, 1 ×  PCR buffer [20 mM Tris-HCl (pH 8.4), 50 mM KCl ], 1.5 mM MgCl_2_, 200 μM dNTPs, 0.5 μM of each primer, and 0.1 μL of Taq DNA polymerase (18038, Invitrogen). The following cycle parameters were used: 3 min at 94 °C, followed by 35 cycles of 94 °C for 45 s, 60 °C for 30 s, and 72 °C for 1 min, and a final extension time of 10 min at 72 °C.

### Deposition of nucleotide sequences

The sequences obtained in this study were deposited in the GenBank short-read archive (SRA), accession number SRR2886919 and 3260963.

## Additional Information

**How to cite this article**: Chen, B. *et al*. Biodiversity and Activity of the Gut Microbiota across the Life History of the Insect Herbivore *Spodoptera littoralis*. *Sci. Rep.* **6**, 29505; doi: 10.1038/srep29505 (2016).

## Supplementary Material

Supplementary Information

Supplementary video S1

## Figures and Tables

**Table 1:** Richness and diversity estimate of the 16S rRNA gene from the pyrosequencing analysis.

Sample	Species richness indices		Species diversity indices	
	Observed	PD tree	Shannon	Simpson
Eggs	43	3	3.10	0.78
Early instar larva	34	3	1.85	0.60
Late instar larva	23	3	1.46	0.50
Pupa	15	1	0.26	0.06
Adult				
Male	46	2	1.71	0.42
Female	73	4	3.03	0.79

PD, phylogenetic diversity.

**Table 2:** The frequency of association of *E. mundtii* in the field-collected *S. littoralis* samples as revealed by diagnostic PCR (*Ent.* = *E. mundtii*).

Stage of development	*n*	*Ent.*Positive	*Ent*. Positive (%)
Egg mass	5	5	100
Larva	10	8	80
Pupa	8	6	75
Adult			
Male	7	5	71
Female	9	8	89
