# Supplementary material for: Biodiversity and Activity of the Gut Microbiota across the Life History of the Insect Herbivore Spodoptera littoralis
Source: Sci Rep. 2016 Jul 8;6:29505. doi: 10.1038/srep29505 (PMC4937375; doi:10.1038/srep29505)

Biodiversity and Activity of the Gut Microbiota across the Life History of the Insect Herbivore *Spodoptera littoralis*

Bosheng Chen[1#], Beng Soon Teh[2], Chao Sun[3#], Sirui Hu[1], Xingmeng Lu[1], Wilhelm Boland[2] and Yongqi Shao[1*]

[1]College of Animal Sciences, Zhejiang University, 310058 Hangzhou, China; [2]Department of Bioorganic Chemistry, Max Planck Institute for Chemical Ecology, Beutenberg Campus, D-07745 Jena, Germany; [3]Institute of Biotechnology, Zhejiang University, 310058 Hangzhou, China.

[*]To whom correspondence should be addressed. E-mail: yshao@zju.edu.cn; Tel./Fax: +86-571-88982305. [#]These authors contributed equally to this work.

Supplementary Video S1 illustrating a typical hatching process of eggs.

Supplementary Table S1 Primers used for the characterization of bacterial taxa

Primer/Probe	Primer sequence (5'-3')	Target	Use
27f	AGAGTTTGATCCTGGCTCAG	Eubacteria	General amplification
4fa	TCCGGTTGATCCTGCCRG	Archaea	General amplification
1492r	GGTTACCTTGTTACGACTT	Eubacteria	General amplification
ITS1	TCCGTAGGTGAACCTGCGG	Fungi	General amplification
ITS4	TCCTCCGCTTATTGATATGC	Fungi	General amplification
M13F	GTAAAACGACGGCCAG	Plasmid	Sequencing
M13R	CAGGAAACAGCTATGAC	Plasmid	Sequencing
968F-GC-Clamp	CGCCCGGGGCGCGCCCCGGGCGGGGC GGGGGCACGGGGGGAACGCGAAGAACC TTAC	Eubacteria	DGGE
1401Ra	CGGTGTGTACAAGGCCCGGGAACG	Eubacteria	DGGE/Sequencing
1401Rb	CGGTGTGTACAAGACCCGGGAACG	Eubacteria	DGGE/Sequencing
968F	AACGCGAAGAACCTTAC	Eubacteria	Sequencing
Gray28F	GAGTTTGATCNTGGCTCAG	Eubacteria	454 sequencing
Gray519r	GTNTTACNGCGGCKGCTG	Eubacteria	454 sequencing

Supplementary Table S2 Bacterial communities in *S. littoralis*

Samples	Total reads	Valid reads	Reads assigned to genus	Phylum	Class	Order	Family	Genus
Egg (D)	2811	2792	1432	5	7	8	10	15
Egg (R)	8001	7903	5471	6	8	10	12	17
E-instar (D)	5517	5509	3595	5	6	7	11	23
E-instar (R)	7905	7797	5093	6	7	10	11	33
L-instar (D)	8771	8614	6869	5	5	6	10	17
L-instar (R)	10855	10801	8820	5	6	6	8	30
Pupa (D)	5271	5260	2511	3	4	5	5	8
Pupa (R)	8329	8290	6801	5	9	14	19	29
Female (D)	3565	3531	3199	3	5	8	12	20
Female (R)	14684	14441	8776	8	14	24	39	62
Male (D)	11905	11841	8653	4	6	7	10	18
Male (R)	30290	30286	22892	3	4	6	10	18

Supplementary Figure S1 Shannon index analysis of the different samples. Shannon indices approach a plateau in each sample.

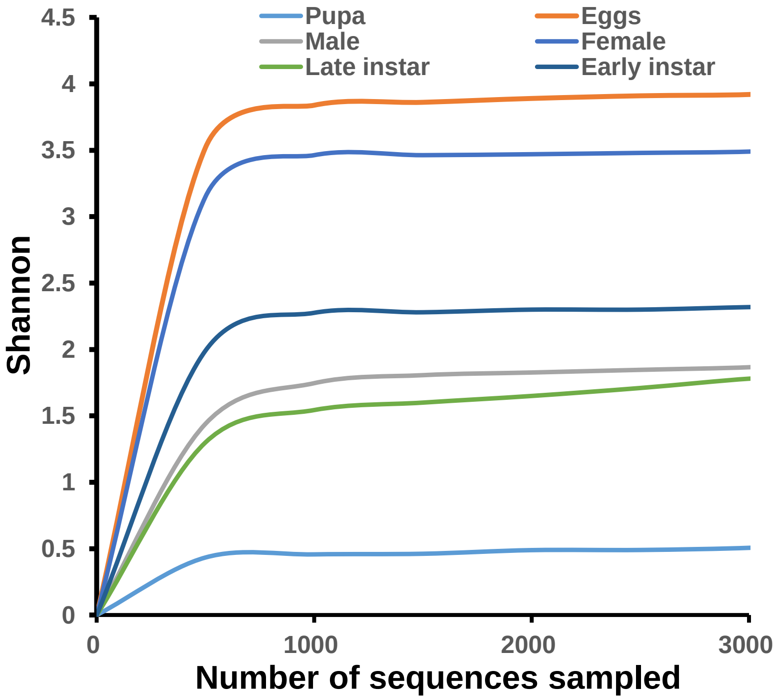

Supplement: Supplementary Information [file srep29505-s1.pdf]
